# Breast Cancer Adjuvant Radiotherapy and Chemotherapy Sequencing: Sequential, Concomitant, or What Else? A Comprehensive Review of the Adjuvant Combinations Journey

**DOI:** 10.3390/jcm13206251

**Published:** 2024-10-19

**Authors:** Grazia Lazzari, Antonietta Montagna, Barbara D’Andrea, Antonella Bianculli, Giovanni Calice, Raffaele Tucciariello, Giovanni Castaldo, Vito Metallo, Giuseppina De Marco, Ilaria Benevento

**Affiliations:** 1 Radiation Oncology Unit, Oncology Research Institute of Basilicata—IRCCS-CROB, 85028 Rionero in Vulture, PZ, Italy; antonietta.montagna@crob.it (A.M.); barbara.dandrea@crob.it (B.D.); giovanni.castaldo@crob.it (G.C.); vito.metallo@crob.it (V.M.); giuseppinademarco.30@gmail.com (G.D.M.); ilaria.benevento@crob.it (I.B.); 2 Medical Physics Department—IRCCS-CROB, 85028 Rionero in Vulture, PZ, Italy; antonella.bianculli@crob.it (A.B.); raffaele.tucciariello@crob.it (R.T.); 3 Laboratory of Preclinical and Translational Research—IRCCS-CROB, 85028 Rionero in Vulture, PZ, Italy; giovanni.calice@crob.it

**Keywords:** adjuvant chemotherapy, radiotherapy, concomitant RT, sequential RT, timing

## Abstract

**Background:** To date, in breast cancer (BC) treatment, adjuvant chemotherapy (A-CT) has preceded adjuvant radiotherapy (A-RT). In the last twenty years, the adjuvant treatment of BC has quickly evolved due to better knowledge of its molecular biology, genetic profile, and α/β ratio of 3/4 Gy for tumor and normal tissue radiosensitivity. Thus, new schedules with hypofractionated radiotherapy have been tested, and a third generation of A-CT has been introduced, raising the question of whether it is time to rethink the sequencing between these two approaches. **Methods:** In the last 20 years, many attempts have been made worldwide to optimize the best sequencing strategy between these two approaches in terms of sequential CT-RT and RT-CT and concomitant and sandwich modalities using drugs and schedules. This paper presents a comprehensive review of the state of the art, analyzing all the available studies to assess the sequencing between A-CT and A-RT with different generations of chemotherapy schedules. **Results:** More than 8000 patients from 30 studies treated with adjuvant chemotherapy and whole breast radiotherapy who were enrolled in randomized, retrospective, and prospective studies were analyzed. Sequential, concomitant, and sandwich modalities of chemotherapy with conventional or hypofractionated RT schedules from the most important studies were included. The most used sequence was adjuvant chemotherapy followed by conventional or hypofractionated radiotherapy. In the concomitant approach, i.v. CMF has been the most important adopted schedule, while the concomitant use of anthracyclines and taxanes with conventional or hypofractionated radiotherapy has been found to be more toxic. One study analyzed the benefit in terms of reducing adjuvant treatment time with upfront hypofractionated radiotherapy and third-generation chemotherapy. **Conclusions:** At present, the best sequencing strategy has not yet been defined. This comprehensive review is a journey among the most important randomized, retrospective, and prospective studies that highlights the past, current, and novel time sequencing proposals between A-CT and A-RT to assess the state of the art and provide useful information for future adjuvant approaches in breast cancer treatment.

## 1. Introduction

### 1.1. Breast Cancer Adjuvant Therapy and “Killing Time” Factor

The standard sequencing strategy in BC adjuvant treatment has been chemotherapy followed by postoperative radiotherapy since the publishing of the Upfront–Outback trial [1,2]. Although data on toxicity, event-free survival, distant metastasis-free survival, and overall survival between the two arms (RT-CT vs. CT-RT) were similar and confirmed in the ten-year update, the CT-RT arm was chosen as the standard approach. The overall adjuvant treatment time has been revolutionized due to novelties in A-CT schedules and A-RT fractionations. With regard to A-CT, the introduction of third-generation chemotherapy combinations has achieved better breast cancer survival with a 20% reduction in breast cancer mortality in comparison with second- and first-generation methods. However, third-generation chemotherapy requires a longer time span to complete the entire course over 12 weeks due to more related toxicities [3]. On the other hand, radiation fractionation schedules have been shortened and are equally effective with longer fractionation regimes, leading to better compliance and quality of life and advantages in patient logistics, waiting lists, and healthcare system costs. In fact, Canadian trials, START trials, Fast Forward trials, and HAI-5 trials all have assured long-term local control and survival rates similar to the standard long-course 2 Gy daily fractionation, with no differences in normal tissue toxicity and better compliance reported by the patients [4]. Thus, is it time to rethink the sequencing strategy? [5].

### 1.2. IORT Boost: A Teaching Lesson on the Role of Killing Time

Radiation starting time is a key factor for local control of cancer [6]. In turn, local control impacts the long-term survival benefit as emphasized by the comprehensive analysis of the complete DBCG 82bc study [7]. How can this happen in BC adjuvant treatment?

It is well-acknowledged that, after surgery, a micro-metastatic migration from the tumor site in the blood may occur as the first step; thus, the tumor bed has been considered a hub for several metastatic processes such as the epithelial-to-mesenchymal transition (EMT) of tumor cells in the vessels [8,9]. Consequently, early local RT is helpful in stopping this migration, as shown by studies conducted on IORT boost models [10]. In fact, data from Belletti et al. have shown the anticancer radiation-induced response of the IORT boost in blocking the proliferation and migration of cancer cells in the fluid taken from the irradiated wound of patients treated with the anticipated boost. To explain this effect, thereafter, they investigated the effects of RT in the modulation of MicroRNAs (miRs) in vivo in human tissues [11]. MiRs are small, non-coding RNA molecules with tissue-specific expression found in animal serum/plasma with the function of post-transcriptionally regulating gene expression [12]. In BC patients treated with lumpectomy and IORT-boost, the analysis of MiR expression profiles in breast tissues from IORT-treated BC patients showed how IORT is capable of modifying the inflammatory wound response through the expression of miR-223 in the peritumoral breast tissue. This response consisted of a downregulation by miR-223 of the local expression of epidermal growth factor (EGF), leading to a decreased activation of the EGF receptor (EGFR) on target cells as a consequence of the positive EGF–EGFR autocrine/paracrine effect induced by the post-surgical wound-healing response. In a second step in the mouse model of BC, both RT-induced miR-223 and peri-operative inhibition of EGFR were able to inhibit the BC cell growth and reduce the recurrence proliferation in the transplanted BC mice. Thus, there is a temporal window from surgery to recurrence in the adjuvant time, in which prompt RT exerts several molecular effects that involve more than simply the direct killing of residual tumor cells. This window still needs to be covered by proper adjuvant sequencing between chemotherapy and radiotherapy.

## 2. The Gap from Surgery to Starting RT

The optimal gap between surgery and adjuvant radiation and chemotherapy has been a controversial matter since the killing time principle was defined by Mackillop WJ in an editorial in 1996 [13]. His famous slogan was “delays in RT should be As Short As Reasonably Achievable” in the ASARA motto, but this target in BC adjuvant therapy has still not been achieved. Looking back, a general perception of the adverse or unrelated survival outcomes between the prolonged interval from surgery and starting RT has grown from different evidence based on studies and populations with heterogeneous chemotherapy schedules (today obsolete), and with this evidence we can draw definitive conclusions on the effective timing of RT.

### Trials and Population-Based Studies: Early or Delayed A-RT?

At the beginning of the 1990s, the general policy in the adjuvant approach was to maximize the use of chemotherapy prior to starting radiotherapy because of several theories on the systemic spread of BC at diagnosis. Thus, chemotherapy should have not only provided a systemic benefit, but should also have induced local control due anti-proliferative effects on any residual disease in the surgical area [14].

However, this was followed by a widespread, disappointing perception that delayed radiotherapy could have a deleterious outcome in high-risk BC patients, which is why several retrospective and then randomized trials were designed on this issue. In a retrospective study conducted on 295 BC patients receiving non-random CMF or anthracycline-based chemotherapy and radiotherapy, the results confirmed this intuition. In fact, in the BC patients who received RT within 16 weeks after surgery, the actuarial 5-year local failure rate was 5% versus 35% in the patients irradiated more than 16 weeks after surgery. The 4-year crude incidences were 4% and 12%, respectively (*p* = 0.06) [15].

In 1993, a retrospective study conducted by Bucholz again assessed that a delay in starting adjuvant RT of over 6 months from diagnosis resulted in a higher local failure rate. Furthermore, an increased rate of distant metastases and a decreased overall survival rate were recorded. In the study, the population was divided into two groups based on the timing of RT delivery. In the early radiation group, BC patients underwent A-RT within 6 months of diagnosis, while in the delayed radiation group, patients received delayed A-RT more than 6 months after diagnosis due to their more intense chemotherapy regimes. The chemotherapy schedules used in both groups were similar. Radiotherapy was delivered with standard fractionation. The best results were recorded in the early radiation group, showing a 98% 8-year actuarial rate for local control vs. 76%, *p* = 0.004; 80% overall survival vs. 52%, *p* = 0.016; and 71% vs. 48% for the disease-free survival, *p* = 0.088. These significant differences were confirmed in the multivariate Cox regression model [16].

Thus, the perception that delayed radiotherapy could have a deleterious outcome in high-risk BC patients raised the need to set up a formal, randomized trial. In the meantime, in 1996, this issue was assessed for the first time in two randomized trials using concurrent CMF in node-positive pre/perimenopausal BC patients (IBCSG trial VI) and in node-positive postmenopausal patients (IBCSG trial VII). The IBCSG VI trial patients were randomized to receive CMF as follows: for either three consecutive courses in months 1–3 or six consecutive courses in months 1–6, both with or without the reintroduction of CMF. In the IBCSG VII trial, patients were randomized to receive tamoxifen for 5 years or tamoxifen for 5 years with three early cycles of CMF, both with or without three courses of delayed CMF. Overall, 718 eligible patients were enrolled: 433 in trial VI and 285 in trial VII. Radiotherapy was provided only on the breast with standard fractionation of 50 Gy and a 10 Gy boost with a delay of 4 or 7 months after surgery for pre/perimenopausal patients and 2 or 4 months after surgery for postmenopausal patients due to CMF completion. As a result, the estimates for the 4-year crude local failures were 8% and 9% (*p* = 0.61) for pre/perimenopausal patients receiving RT at 4 or 7 months after surgery and 3% and 6% for postmenopausal patients treated with RT at 2 months or 4 months after surgery (*p* = 0.63). Thus, a modest delay of RT of 4 to 7 months following breast-conserving surgery and CMF through these two randomized trials seemed acceptable regarding the risk of worst outcomes [17].

Years later, van Maaren Marissa C et al. analyzed the impact on 10-year disease-free survival (DFS) in a Dutch BC population-based cohort treated with adjuvant therapy according to Dutch guidelines, which advised starting radiation therapy (RT) within 5 weeks after breast-conserving surgery (BCS). They identified a population named group 2, which included patients treated with chemotherapy, and compared chemotherapy before (BCS–chemotherapy–RT) and after RT (BCS–RT–chemotherapy). As a result, the timing did not affect survival in BCS–chemotherapy–RT. In BCS-RT–chemotherapy, distant metastases-free survival was higher for >55 days than the <42-day interval [18]. The implications of these results are still debated. Recently, an Ontario population-based study on 1028 women with stage I–II BC treated with adjuvant RT-CT focused on the impact related to the delayed start of A-RT. As a result, a delay in RT of over 12 weeks from surgery or 6 weeks from the end of CT was related to a worse survival probability [19]. In the RT-only group, a waiting time of 12 weeks or more from surgery to the start of radiation was associated with worse event-free survival after a median follow-up of 7.2 years (HR: 1.44; 95% confidence interval: 0.98 to 2.11; *p* = 0.07). In the group that received intervening adjuvant chemotherapy before RT, a waiting time of more than 6 weeks from completion of chemotherapy led to a worse event-free survival after a median follow-up of 7.4 years (HR: 1.50; 95% confidence interval: 1.00 to 2.22; *p* = 0.047). Looking back, the Upfront–Outback trial warned in its concluding paragraph to rethink the sequencing between A-CT and A-RT in case of prolonged chemotherapy schedules [2], which is especially pertinent today. In this comprehensive review, we will detail the most important studies assessing the best sequencing using the past and modern CT-RT combinations in terms of efficacy, toxicities, and total adjuvant duration time in order to determine whether there is an optimal killing time in the era with the new, standard, third-line A-CT and HF-RT schedules.

## 3. First–Second-Generation Chemotherapy and Conventional Fractionated Radiotherapy

### 3.1. Chemotherapy and Sequential Radiotherapy vs. Radiotherapy and Sequential Chemotherapy

A-CT followed by A-RT still remains the standard of care. This approach was the first attempt to combine adjuvant chemotherapy and radiotherapy, starting from the results of the trial published in 1996 and then updated in 2005. In the study, 244 patients were randomized to receive CT before RT vs. RT before CT [1,2]. Chemotherapy consisted of 12 weeks of therapy with four cycles of CAMPF repeated every 21 days. Radiotherapy consisted of 25–30 fractions with standard fractionation on the breast and nodal areas. In the first report of 1996, no differences in the 5-year actuarial rates of cancer recurrence at any site and of distant metastases in the RT-first group and the CT-first group (*p* = 0.17 and *p* = 0.05, respectively) were found. No differences in toxicity or in overall survival (*p* = 0.11) occurred. The update at ten years confirmed these previous data showing no statistically significant difference in the rates of freedom from any event, freedom from distant metastasis, or overall survival and toxicity between the CT-first and RT-first arms. However, A-CT before A-RT was chosen in the clinical practice assuming that delayed radiation after 12 weeks of chemotherapy from surgery could not have a negative impact on survival and local control outcomes. In the conclusions, the authors could not extrapolate these results to longer chemotherapy regimens. Interestingly, the median interval between surgery and the start of RT was 36 days (5 weeks) in the RT-first group and 126 days (31 weeks) in the CT-first group. A gap of more than 16 weeks was recorded between the surgery and RT in 1% of the RT-first group and 84% of the CT-first group. The median interval from the first breast excision to the start of CT was 29 and 13 weeks in the RT-first and CT-first groups, respectively. The time taken to start CT was less than 16 weeks from surgery. See Table 1.

### 3.2. Concurrent Chemotherapy vs. Sequential Radiotherapy

In these studies, due to the advances in novel drug discovery at the end of the 1990s, schedules containing mitoxantrone, taxanes, anthracyclines, and the most used CMF were delivered concurrently or sequentially with standard fractionated RT. This indicates the advantages of the concomitant arm in terms of local control with several differences in toxicity.

A first study on concurrent treatment was proposed by Serin et al. using an anthracenedione agent like mitoxantrone to reduce cardiac toxicity [20]. In the study, 154 patients affected by stage I or II BC received conventional RT of 50 Gy in 25 fractions concurrently with chemotherapy. Chemotherapy consisted of 5-fluorouracil, mitoxantrone, and cyclophosphamide every 21 days for four to six cycles. G1 cutaneous toxicity occurred in 62.3% of the cases, and severe G3 radiation dermatitis requiring temporary interruptions of therapy was found in 4.5% of patients. Only one case of G3 acute cardiac toxicity was recorded.

Bellon et al. presented a retrospective study on 45 high-risk breast cancer patients treated with RT concurrently with taxanes (paclitaxel or docetaxel) [21]. Radiation was delivered to the chest wall or breast with doses ranging from 4680 cGy to 5040 cGy. A boost dose of radiation was provided to most of them, while nodal RT was delivered when indicated. Severe acute skin toxicity was 20% in the docetaxel arm vs. the paclitaxel arm (*p* = 0.04). With a median follow-up of 11 months, only one patient developed skin late toxicity. The use of taxanes concurrent with A-RT was reported by Burnstein in a randomized trial on 40 BC patients [22]. Paclitaxel was delivered according to two schedules: 60 mg/m^2^ weekly × 12 weeks or 135–175 mg/m^2^ every 3 weeks × 4 cycles. As a first result, weekly paclitaxel treatment at 60 mg/m^2^ per week with concurrent radiation was the dose-limiting toxicity in 4 of 16 patients (25%), with one case of severe pneumonitis requiring steroids. To the contrary, dose-limiting toxicity was not recorded among patients receiving concurrent radiation with paclitaxel given every 3 weeks at 135–175 mg/m^2^. However, G2 radiation pneumonitis not requiring steroid therapy was seen in 2 of 24 patients (8%). No severe radiation dermatitis was observed in either of the paclitaxel schedules. The authors concluded that, while concurrent treatment with weekly paclitaxel and radiation therapy was not advisable, the concurrent arm with the less frequent paclitaxel dosing schedule was determined to be less toxic, although there was the possibility of pulmonary injury as a side effect.

In the set of anthracycline, a dose-dense intensification study with standard fractionated RT given sequentially or concomitantly was assessed by Sanguineti et al. [23]. The study provided a mild RT dose intensity (DI) to compensate gaps with 2.3 Gy per fraction in case of RT interruptions for toxicity. Forty-seven stage I–II breast cancer patients, after conserving surgery, were randomized to receive the CEF regimen delivered every two weeks (CEF14) or three weeks (CEF21). RT was applied to the residual breast with a total dose of 50 Gy in five weeks. Radiotherapy DI was expressed as the average total dose received each week, defined as the “weekly dose-rate” (WDR). As a result, 98.8% of patients received a cumulative total dose of RT within ±10% of that planned. The type of treatment (CEF14 vs. CEF21) did not affect the probability of WDR < 9.5 Gy/wk. Regarding the CT-RT overlap, patients receiving more than two cycles of chemotherapy during radiotherapy had an increased risk of RT delay compared to other patients (RR = 3.74, 95% CI: 1.44–9.48, *p* = 0.0063).

Rouëssè J et al. enrolled 638 BC patients in a randomized trial providing two chemotherapy arms concurrent with or sequential to standard fractionated radiotherapy [24]. The A arm consisted of 5-fluorouracil 500 mg/m^2^, mitoxantrone 12 mg/m^2^, and cyclophosphamide 500 mg/m^2^ and concurrent RT. In the B arm, adjuvant CT with the FEC regime and sequential standard RT were provided. Chemotherapy was administered on day 1 every 21 days for four cycles. The median treatment duration times were 64 days in the A arm and 126 days in the B arm. No significant difference in overall or disease-free survival was observed, but a 5-year locoregional relapse-free survival of 3% in arm A and 9% in arm B (*p* = 0.01) was recorded. The risk of locoregional recurrence in the B arm was increased 2.8-fold according to multivariate analysis (*p* = 0.027). In the A arm, the most frequent acute toxic effect was febrile neutropenia with grade 3–4 leukopenia. At 1 year, cardiac side effects like subclinical left ventricular ejection fraction events were reported due to concomitant radiotherapy (*p* = 0.02).

In the ARCOSEIN trial, 716 patients were randomized to receive CT and sequential RT versus concurrent RT using a CT regimen with mitoxantrone 12 mg/m^2^, 5-fluorouracil 500 mg/m^2^, and cyclophosphamide 500 mg/m^2^ every 21 days for six cycles and standard fractionated RT. Adjuvant treatment started within 6 weeks after surgery. As a result, the 5-year DFS was 80% in both groups (*p* = 0.83). Moreover, no statistically significant difference in locoregional recurrence-free survival or OS was observed (*p* = 0.76). However, in the node-positive subgroup, the 5-year LRFS was improved in the concurrent arm (*p* = 0.02), corresponding to a decreased risk of locoregional recurrence by 39% (HR, 0.61; 95% CI, 0.38 to 0.93). Moderate, acute, locoregional toxicities were found in the two arms [25].

Two concomitant AC-based regimes with standard RT study were conducted by Livi et al. on 60 BC patients. Four cycles of AC (doxorubicin plus cyclophosphamide) vs. four cycles of epirubicin (EPI) followed by four courses of i.v. CMF every 28 days were adopted. RT was applied to the breast and nodal areas with a 50 Gy mean delivered dose (range 46–52 Gy). The boost dose was 10 Gy for patients with tumor-free surgical margins and 20 Gy for patients with positive margins. As a result, the concomitant treatment yielded acute skin G3 toxicity in 8.9% of patients, with one case of G4 toxicity (1.7%). RT was stopped in 21.3% of patients with a temporary RT delay of a mean of 5 days. A CT interruption of ≤7 days was applied in 57.1% of patients because of hematological toxicity. An asymptomatic reduction in the left ventricular ejection fraction of >10% and <20% of the baseline value was recorded in both groups [26] (see Table 1).

### 3.3. CMF or AC (Concurrent vs. Sequential RT)

The CMF combination has been the most used treatment delivered concurrently with RT over the AC schedule. Faul et al. tested the efficacy of concurrent “standard” CMF vs. i.v. CMF regimes in 116 BC patients with stage I-II BC treated with CMF and A-RT [27]. The standard CMF regime consisted of cyclophosphamide 100 mg/ m^2^ on days 1–14, methotrexate 40 mg/m^2^ i.v. on days 1 and 8, and 5-fluorouracil 600 mg/m^2^ on days 1 and 8 repeated every 28 days for six cycles. The i.v. CMF regimen consisted of cyclophosphamide 600 mg/m^2^ i.v., methotrexate 40 mg/m^2^ i.v., and 5-fluorouracil 600 mg/m^2^ i.v. given every 21 days for six to eight cycles. Radiotherapy was delivered according to standard fractionation. In the study, 73 patients were treated prospectively with concurrent therapy and were retrospectively compared with a matched group of 40 patients treated with sequential or sandwich therapy. Concurrent sequencing did not influence the scheduled delivered RT and CT doses. No significant differences in acute skin reactions or complications between the two groups were recorded with a small and significant delay in RT delivery: 1.32 days (0–15) vs. 0.36 (0–7) in the concurrent group (*p* = 0.03). Sequencing had no significant impact on hematological toxicity. “Standard” CMF impacted treatment delivery more than i.v. CMF. In fact, the RT delay was 2.2 days versus 0.26 (*p* = 0.002), and the percentage of delivered chemotherapy was 93% vs. 99% (*p* = 0.000004). The study showed that i.v. CMF was safe when delivered concurrently with standard RT.

To confirm the safety of concurrent administration of CMF vs. an anthracycline-based regimen, Fiets et al. reported on a prospective, non-randomized, comparative study on the acute toxicity of RT alone vs. RT given concurrently (RCT) to doxorubicin-cyclophosphamide (AC/RT) or CMF (CMF/RT). Standard fractionated RT on breast, chest wall, and nodal areas was applied [28]. In total, 112 patients received CT/RT as follows: 61 patients were treated with AC/RT and 51 with CMF/RT; forty-two patients were treated with RT only as controls. As a result, patients treated with AC/RT and CMF/RT had significantly higher incidences of high-grade toxicity compared with those treated with RT only. The AC/RT scheme was more associated with significant, high-grade skin toxicity than CMF/RT. Less than 85% of the planned chemotherapy reduction dose was applied to 11% of patients, and 17% of patients treated with RCT had an admission to hospital. The authors concluded that RCT administration yielded an unacceptably high level of acute toxicity, mainly with the AC regimen.

Arcangeli et al. published results of a randomized trial on RT concomitant vs. sequential i.v. CMF for six cycles conducted on 206 eligible patients [29]. A-RT was applied only to the whole breast at a dose of 50 Gy in 20 fractions followed by an electron boost of 10–15 Gy in the tumor bed. Patients in the concurrent treatment group generally received one cycle of CMF before starting the RT course. Radiation therapy started within 2 months from the day of definitive surgery and concomitantly with the second CMF course in the concurrent treatment group. In the sequential treatment group, RT started 7 months after surgery, mainly at the conclusion of the last CMF course. All patients in the two groups received 100% of the planned dose. There were no RT breaks during the radiation time for skin or hematologic toxicity. All patients completed the planned radiotherapy and chemotherapy. No differences in 5-year breast recurrence-free survival (*p* = 0.97), metastasis-free survival (*p* = 0.44), disease-free (*p* = 0.99), or overall (*p* = 0.56) survival were observed in the two treatment groups. No increased risk of toxicity was observed between the two arms. The authors concluded that i.v. CMF concurrent with RT, due to its advantage in shortening the overall treatment time, could have been more useful to patients with a high risk of recurrence, like those with R1 surgical margins and a large tumor size.

In the prospective study of Han et al., 238 patients with stages I and II breast cancers were prospectively enrolled in a study with i.v. CMF concurrent vs. sequential RT [30]. After BCS, all patients underwent i.v. CMF every 3 weeks for six cycles. RT on breast and nodal areas was delivered with standard fractionation with a 56 Gy median dose. In the sequential group, RT was started after the completion of three cycles of CT; then an additional three cycles of CT were delivered after RT. There was no significant difference in G3 or G4 hematologic toxicities during CT between the two groups, and no difference in RT-related adverse effects was observed. During the median follow-up of 42 months, systemic recurrences occurred in 18 patients (13.5%) of the concurrent group and in 20 patients (19.1%) of the sequential group.

Livi et al. in the same year showed a retrospective three-arm study comparing 485 patients treated with conservative breast surgery, six courses of CMF, and postoperative whole-breast RT vs. 300 patients who received postoperative CMF only vs. 509 patients treated with postoperative whole-breast RT only [31]. As a result, G2 acute skin toxicity occurred in the concurrent group (21.2% vs. 11.2% of the RT-only group, *p* < 0.0001). The local recurrence rate was 7.6% in the CT/RT group and 9.8% in the RT group; this difference was not statistically significant in univariate analysis (log-rank test *p* = 0.98). However, in a multivariate analysis that was also adjusted for pathological tumor, pathological nodes, and age, the CT/RT group showed a statistically lower rate of local recurrence (*p* = 0.04).

A retrospective study on 244 BC patients treated with radical surgery or breast conservative surgery [32] was conducted by Ismaili et al. In the study, two adjuvant schedules of chemotherapy concurrently given with radiotherapy were compared. In the A group, 110 BC patients received anthracycline-based chemotherapy according to several regimens. All cycles were delivered every 21 days for six courses: AC 60 (doxorubicin 60 mg/m^2^ and cyclophosphamide 600 mg/m^2^); FEC75 (5-fluorouracil 500 mg/m^2^, epirubicin 75 mg/m^2^, and cyclophosphamide 500 mg/m^2^); and i.v. FAC50 (5-fluorouracil 500 mg/m^2^, doxorubicin 50 mg/m^2^, and cyclophosphamide 500 mg/m^2^). The B group consisted of 134 BC patients receiving i.v. CMF every 21 days for six courses. Conventional fractionated radiotherapy of 50 Gy on the whole breast (or on the external wall) and/or on the nodal areas was provided. As a result, after 76.4 months median follow-up, locoregional relapse occurred in one patient in the anthracycline-treated group and in eight patients in the i.v. CMF-treated group. The disease-free survival and overall survival after 5 years were not statistically significant between the two groups (*p* = 0.136 and *p* = 0.428, respectively). However, the locoregional disease-free survival at 5 years was better in the A group than the B group (98.6% vs. 94% *p* = 0.033). The rate of G2 and G3 anemia was 13.9% and 6.7% in anthracycline group and CMF group, respectively (*p* = 0.009). Grade 2–3 skin toxicity was 4.5% in the A group vs. 0% in the B group B (*p* = 0.013). Thus, anthracycline treatment concurrent with RT was more effective and toxic (see Table 2).

## 4. Third-Generation Chemotherapy and Conventional Fractionated Radiotherapy

In this section, the long-course chemotherapy regimens using combinations of anthracycline and taxane schedules reflect the routine daily practice. Radiotherapy is still delivered with standard fractionation.

### 4.1. Concurrent Chemotherapy vs. Sequential Radiotherapy

In a randomized, controlled trial by Ying et al., 155 locally advanced BC patients were treated with an FEC chemotherapy regimen followed by docetaxel (FEC-D) after radical mastectomy and then randomized to concurrent or sequential A-RT [33]. Patients randomized in the concurrent (CCRT) group were treated with docetaxel. In the second arm, radiotherapy was delivered after chemotherapy (SCRT). At 39 months median follow-up (range 16–62), the 3-year local–regional recurrence-free survival in the CCRT group was 92.3% vs. 81.8% in the SCRT group (*p* = 0.046). There was no significant difference in 3-year disease-free survival and overall survival: 76.9% and 64.9% (*p* = 0.073) and 87.2% and 81.8% (*p* = 0.342), respectively. The CCRT sequence yielded better outcomes in the pT3–4 pN1–3 cM0 subgroups. In fact, the 3-year local recurrence-free survival was 88.2% vs. 69.4% in the SCRT arm (*p* = 0.036). The DFS rate was 72.6% in the CCRT group vs. 41.7% (*p* = 0.049). No significant difference was observed in OS (79.4% vs. 69.4%, *p* = 0.313). No differences were recorded in terms of hematologic and skin toxicities; there was a G3–4 leukopenia in the CCRT group of 29.5% vs. 31.2% in the SCRT arm (*p* = 0.858). The prevalence of grade 1–2 radiation skin reactions was 89.7% in the CCRT arm vs. 88.3% in the SCRT arm (*p* = 0.548).

A prospective feasibility study by Ali Assan et al. was published on 43 BC patients treated with modified radical mastectomy or breast conservative surgery and long-course adjuvant chemotherapy concurrent with RT [34]. Adjuvant chemotherapy consisted of four cycles of AC followed by four cycles of paclitaxel 60 mg/m^2^ weekly for 12 weeks and concurrent RT with standard fractionation. As a result, at 36 months median follow-up, the overall survival was 95%, and disease-free survival was 92.5%. No local relapse or radiation pneumonitis was assessed. However, a delay in starting radiotherapy was recorded for 15% of patients because of acute G3 skin toxicity (10%) or severe mucositis and wound gap (2.5%). At the last follow-up, the cosmetic outcome score was found to be excellent in 62.5% of the patients.

### 4.2. Sandwich Modality of CT-RT vs. Sequential RT

Woo J et al. reported on a retrospective study with sandwich CT sequencing with radiotherapy [35]. In the study, 200 BC patients with node-positive disease were treated with four cycles of AC chemotherapy followed by taxanes as follows: four cycles of paclitaxel 175 mg/m^2^ or docetaxel 75 mg/m^2^ every 3 weeks. The sandwich group consisted of 110 BC patients, while 90 patients were treated as a control group that received RT after the whole CT course (four weeks after the last CT drug administration). In the sandwich RT group, RT started four weeks after the last AC cycle. The courses of paclitaxel or docetaxel were administered within three weeks of completion of RT. The dose of irradiation was 50.4 Gy for the whole breast or chest wall and nodal areas. A 10 Gy boost on the tumor bed was provided. The gap between surgery and starting RT was 3.0 ± 0.8 months in the sandwich group vs. 5.5 ± 1.2 months in the control group. At a mean of 105.4 months of follow-up time, the locoregional recurrence rate was 3.6% in the sandwich RT group vs. 12% in the control group (*p* = 0.012); there was no significant difference in distant metastasis between the two groups (25% vs. 17%, *p* = 0.220). In the multivariate analysis, the RT sequencing appeared as an important predictor factor for LRR (*p* = 0.017). Moreover, in the luminal A subtype, DFS improved vs. non-A luminal subtypes (*p* = 0.001 vs. *p* = 0.670) in the sandwich RT group vs. the control group. The sequencing did not impact OS differences between A luminal and non-A luminal subtypes (*p* = 0.150 vs. *p* = 0.679).

## 5. Chemotherapy and Sequential Hypofractionated Radiotherapy

This approach is characterized by the introduction of hypofractionated radiotherapy (HF-RT) with several schedules varying from 16/15 to 5 fractions with or without a boost (sequential or concomitant). In most of these studies, patients received second- or third-generation chemotherapy schedules prior to RT. Recall that, initially, the use of HF-RT after adjuvant chemotherapy in 2011 was not advised by the ASTRO consensus, due to several uncertainties of unknown or unexpected toxicities [36]. Finally, in 2018, ASTRO guidelines provided no limitations to its use with any kind of adjuvant chemotherapy [37]. All studies on HF-RT have included patients treated with chemotherapy and sequential RT at least two weeks after chemotherapy.

### 5.1. First–Second-Generation Chemotherapy Followed by Sequential, Moderate, Hypofractionated Radiotherapy: The Hypofractionated Trials

In Ontario’s randomized trial, 622 patients received the experimental arm consisting of 266 cGy/fraction × 16 fractions on the whole breast using the 2–3D technique. In this arm, 70 patients received A-CT consisting of CMF given before RT. The use of systemic therapy did not affect outcomes in terms of survival and toxicity with the control arm, as shown in the following 10-year update in 2010 [38].

The following UK trials tested three schedules of hypofractionated radiotherapy on the breast and supraclavicular area, all of them after completion of A-CT. In START trial A, 737 patients were randomized to receive 39 Gy in 13 fractions, while 750 patients received 41.6 Gy in 13 fractions. Overall, 534 patients received A-CT according to the current guidelines. RT started with a gap of two weeks after chemotherapy. The anthracycline-containing regimen was prescribed in 70.1% of patients for the group receiving 41.6 Gy and in 71.1% for the 39 Gy arm. Intravenous CMF was prescribed in 29.5% for 41.6 Gy and 26.7% for the 39 Gy arm. Adjuvant taxanes were delivered to nine patients in the 41.6 Gy arm and four patients in the 39 Gy arm [39]. In START trial B, 1110 patients were allocated to the experimental arm and received 40 Gy in 15 fractions. In this arm, A-CT was delivered in 233 patients, with A-RT delivered with a gap of two weeks after chemotherapy. The anthracycline-containing regimen was delivered in 58.4% of patients. CMF combination therapy alone was prescribed in 39.9% of patients. Only six patients allocated to this arm received adjuvant taxanes [40]. In these three trials, the outcomes of sequencing with adjuvant chemotherapy were not the end-point.

Deantonio et al. proposed a randomized study enrolling 85 patients treated with 2.25 Gy/fraction for 20 fractions to a total dose of 45 Gy, followed by a boost dose of 9 Gy in three fractions for the tumor bed vs. the standard fractionated RT [41]. Upfront chemotherapy with CMF, EC, and FEC was provided with hormone therapy given concomitantly with radiotherapy. The median treatment time spent was 29 days for HF-RT and 37 days for the conventional radiotherapy. RTOG acute skin toxicities in erythema were recorded in 85% of BC patients treated with hypofractionation and in 96% of patients treated with conventional fractionation (*p* = 0.01). In the logistic regression model, neither chemotherapy nor breast volume and breast maximum dose had an impact on toxicity. After 6 months, late toxicity was 10% in the hypofractionation group and 15% in the conventional fractionation group (*p* = 0.4), and these results were not statistically significant according to the Kaplan–Meyer method (*p* = 0.17). The risk of late toxicity at 12 months was 5.9% and 29.2% in the group treated with hypofractionation; this risk at 30 months was 8.2% and 10.6% in the group treated with standard RT, respectively.

Hijal T et al. showed results of a retrospective study in which 162 patients were treated with hypofractionated RT with or without adjuvant CT regimens with anthracyclines and taxanes. Radiotherapy consisted of 42.4 Gy in 16 fractions on the breast. The chemotherapy was delivered in 48% of the studied BC patients; this group received a boost to the tumor bed [42]. Grade 3 acute skin toxicity or higher was similar between the two groups: 2.1% in the chemotherapy group vs. 4.4% in the radiation-alone group (*p* = 0.67). Grade 0 acute skin toxicity was not statistically different (*p* = 0.67). At 16 months minimum follow-up, late skin toxicity was not significant. Late G3 or higher skin toxicity was 2.1% in the chemotherapy group vs. 0% in the no-chemotherapy group (*p* = 0.30). Excellent or good scores for cosmetic outcomes were similar in both groups (*p* = 0.80).

Kouloulias et al. retrospectively analyzed 116 breast cancer patients with T1-2 N0 Mx treated with 3D conformal radiotherapy with a total dose of 50.54 Gy or 53.2 Gy in 19 or 20 fractions according to stage over 23–24 days with the last three to four fractions as a sequential boost [43]. Two chemotherapy schedules were used: EC followed by taxanes and CMF. The anthracycline regimen consisted of EC for four cycles every 2 weeks (wks), a 3-week break, and then four cycles of taxotere every 3 wks. The second group received six cycles of CMF chemotherapy every 21 days. The EORTC/RTOG acute skin toxicity was not significant for chemotherapy (*p* = 0.154) in either of the radiotherapy fractions (*p* = 0.47). Although in univariate analysis only chemotherapy was significantly related to the acute skin toxicity with *p* = 0.05, in the multivariate analysis, this effect was not confirmed. None of the patients during the 2 years of follow-up presented any locoregional relapse. In conclusion, any type of chemotherapy combined with hypofractionated RT had a negative impact on acute skin toxicity.

These results were confirmed by De Sanctis et al. in a prospective study on 510 patients treated with a chemotherapy regimen consisting of three cycles of Adriamycin 60 mg/m^2^ and three cycles of Taxol 200 mg/m^2^ followed by three cycles of CMF and sequential HF-RT with or without a boost according to Ontario or UK-START trials [44]. The mean gap time between chemotherapy and radiotherapy was 2 months (range 6 days–3.8 months). No treatment interruptions were recorded for acute toxicity. The prevalence of acute skin toxicity G1 was 61.3%, while that of G2/G3 was 20.5% and that of G2/G3 was 4.3%. In the univariate analysis, significant factors related to late fibrosis were chemotherapy (*p* = 0.04), diabetes (*p* = 0.04), and boost administration (*p* < 0.01), but in the multivariate analysis, these effects were not confirmed. Only the boost administration was related to the skin effect (*p* = 0.02).

In a single-arm clinical trial by Vijayaraghavan et al., 67 patients after modified radical mastectomy were treated with anthracyclines plus taxanes followed by adjuvant HF-RT with a VMAT or IMRT modality. Treatment resulted in grade 2 and higher acute radiation dermatitis in 11.9% patients, with one patient developing a grade 3 skin toxicity [45]. With a median follow-up of 9 months, there were no significant late toxicities.

### 5.2. Second–Third-Generation Chemotherapy Followed by Ultra-Hypofractionated Radiotherapy

Hans Van Hulle et al. conducted two prospective clinical trials on a new, accelerated, fractionated radiation schedule in NCT04098926 and NCT03121248 [46,47]. In both studies, more than 150 BC patients were treated with hypofractionated RT consisting of five fractions of 5.7 Gy to the whole breast or chest wall with a 28.5 total dose (TD), 5.4 Gy on regional nodes with a 27 Gy TD and a simultaneous boost (SIB) of 6.2 Gy or 6.5 Gy (31 Gy and 32.5 Gy) when required. Chemotherapy always preceded RT with a minimum gap of 3 weeks. Patients received EC for four cycles followed by 12 cycles of paclitaxel and hormone therapy. As a result, the role of adjuvant chemotherapy was not an influencing factor on acute and late toxicity.

In recent years, the multicenter, phase 3, randomized, non-inferiority trial FAST-Forward was published during the COVID-19 pandemic. In this trial, a total of 4096 BC patients were enrolled. The trial provided three RT arms: 40 Gy in 15 fractions (over 3 weeks), 27 Gy in five fractions (over 1 week), and 26 Gy in five fractions (over 1 week) to the whole breast or chest wall. Nodal areas were also included. More than 1000 patients received adjuvant chemotherapy before radiotherapy. Chemotherapy schedules consisted of anthracyclines or taxane regimens as per guidelines. No differences in acute and late skin toxicity rate were recorded among the arms [48].

## 6. Concurrent Hypofractionated Radiotherapy and Chemotherapy

### 6.1. Concurrent vs. Sequential CMF and Moderate Hypofractionated RT

Isaac et al. reviewed the records of BC patients who received adjuvant CMF, most of them with hypofractionated RT. In this analysis, 202 BC patients were treated with concurrent HF-RT, while 47 patients received a sequential schedule of CMF/HF-RT. Mastectomy was allowed in 64% of patients, and nodal involvement occurred in 88% of them [49]. The HF-RT schedule was 2.5 Gy/f to 40.0 Gy in 16 fractions, without a boost. RT interruption or discontinuation for acute toxicity occurred in 4% of patients in the concurrent arm vs. 0% in the sequential (*p* = 0.36), with G3 or G4 RT-induced toxicity in 1.5% and 2.1% for the concurrent and sequential groups, respectively (*p* = 0.57). The median relative dose intensity of chemotherapy for patients receiving concurrent CMF/RT was 0.87; for those receiving sequential CMF/RT, it was 0.84, while in the CMF group alone, it was 0.85 (*p* = 0.22). The authors concluded that CMF given concurrently with moderate hypofractionated RT had a low risk of severe toxicity, and it was considered a good choice of adjuvant regimen.

### 6.2. Concurrent Third-Generation Chemotherapy to Moderate Hypofractionated Radiotherapy

Chen et al. defined a phase II trial on HF-RT with concurrent paclitaxel, enrolling 44 patients with stage II or III breast cancer [50]. All had received breast-conserving surgery and four cycles of AC with a further four cycles of paclitaxel 175 mg/m^2^ every 3 weeks. Radiotherapy was delivered concurrently during the first two cycles of paclitaxel using 39.6 Gy TD in 22 fractions and a tumor bed boost of 14 Gy in seven fractions. As a result, the 5-year actuarial rate of disease-free survival was 88%, and overall survival was 93%. At 75 months median follow-up, no radiation pneumonitis occurred. No significant changes in the diffusing capacity for carbon monoxide (DLCo) soon after radiotherapy (*p* = 0.51) or in the following follow-up (*p* = 0.63) were recorded. The acute cosmesis score was related to the volume of irradiated breast tissue. Only two patients developed acute grade 3 skin toxicity.

In the CONCERT study, 60 patients with stage II–III invasive BC were treated with adjuvant taxane-based chemotherapy and HF-RT 40 Gy in 15 fractions ± boost [51]. Regional RT was allowed without the internal mammary chain. RT started with the third cycle of adjuvant taxanes given in a 3-weekly schedule or with the eighth cycle in a weekly schedule. Adjuvant taxane CT was delivered in four cycles of 3-weekly paclitaxel 175 mg/m^2^ or 12 cycles of weekly paclitaxel 75 mg/m^2^ after anthracyclines. All HER2-positive patients received adjuvant trastuzumab, while in ER/PR-positive BC patients, standard endocrine therapy was provided. As a result, 36 patients received the 3-weekly paclitaxel regimen, and 24 received the weekly paclitaxel regimen. No G3 or G4 toxicity was recorded with any treatment interruption or CT-RT program completion in all treated patients. The median ejection fraction pre- and post-6 months CT-RT was 60% (*p* = 0.177). No cardiac or pulmonary disfunctions occurred. At a median follow-up of 34 months, the 3-year actuarial rate of disease-free survival and overall survival was 75% and 98.3%, respectively. Due to an acceptable toxicity profile, the author concluded that concurrent adjuvant chemoradiotherapy in breast cancer should have been considered in order to shorten the overall treatment time with the benefit of cost-effectiveness.

## 7. Back to the Future: Moderate and Ultra-Hypofractionated Radiotherapy and Sequential Third-Generation Chemotherapy

Inspired by the Upfront–Outback trial [1,2], a novel approach using hypofractionated radiotherapy before third-generation chemotherapy was outlined in a retrospective study published in 2024. Data on acute–subacute toxicities and on the reduced gap in the overall adjuvant treatment time have been provided.

### Upfront Hypofractionated Radiotherapy Before Third-Generation Chemotherapy

In this retrospective study, 45 BC patients were treated with third-generation A-CT after hypofractionated RT in 15–5 fractions in the IMRT or VMAT modality with or without a sequential or simultaneous integrated boost (SIB) [52]. Upfront hypofractionated RT was adopted according to the HY5 protocol and START B trial. AH-RT started 35 median days (5 weeks) after surgery (30–45 days). Third-generation A-CT was delivered as per clinical guidelines and started within a mean of 10 days (8–15 days) after RT. The median interval from surgery and chemotherapy was 8 weeks (6–12 weeks). The median duration for the entire adjuvant treatment was 35 weeks from the surgery (26–40 weeks), according to the RT and CT schedules. No differences in G2–G3 acute skin and lung toxicities between the two AH-RT arms and CT schedules were found in univariate and multivariate analyses (*p* = 0.077 and *p* = 0.68; 0.67 and 0.87, respectively). At 6 months, the cumulative incidence of G3 skin toxicity was 3 for AH-RT—5 fractions—and 2.5% for AH-RT—15 fractions (*p* = 0.672). Lung toxicity was 3.2% and 2.5% (*p* = 0.618), respectively. These results again propose a new randomized trial to assess the sequencing of ultra- and moderate hypofractionated RT, which has demonstrated several advantages in terms of logistics, waiting lists, and healthy system costs.

## 8. Conclusions

Since 1991, the issue of the best sequencing between adjuvant chemotherapy and radiotherapy in breast cancer has been focused on in many studies, with the aim of optimizing the overall adjuvant treatment time, reducing toxicity, and maximizing the benefits in local control and disease-free survival. Retrospective, prospective, and randomized studies across the last 25 years been conducted to determine the best approach according to the available drugs and radiation knowledge of the faculties. Schedules with AC alone or CMF have been replaced with more effective and complex combinations using anthracyclines followed by taxane schedules that delay the start of RT beyond 12 weeks. According to the data provided in the reported studies, this delay seems acceptable when considering the upfront first–second generation of CT, but the impact of third-generation chemotherapy in the delay to RT is still unknown and again raises the same doubts as those of Bucholtz twenty years ago [16]. Fractionated radiotherapy has completely changed, due to a better comprehension of the α/β ratio for the tumor and the normal breast tissue. At present, moderate hypofractionated radiotherapy in 15–16 fractions is considered the standard fractionation. Ultra-hypofractionation in five fractions is expected to replace this method soon. The use of a simultaneous boost (SIB) with IMRT modalities is also a new attempt to shorten the RT delivery [53]. However, radiotherapy still follows chemotherapy, while the concomitant approach is less valued. Thus, considering the advantage of upfront HF-RT in reducing the gap from surgery, the initiation of chemotherapy, and the overall adjuvant treatment time, the benefit for BC survival outcomes is ensured in line with the ASARA principle and the IORT boost, as shown by the experiments of Belletti and Iorio [10,11,12]. Is it time to rethink the sequencing? This remains a work in progress.

## Figures and Tables

**Table 1:** First–second-generation chemotherapy and conventional fractionated radiotherapy.

Authors	Study	Sequencing	Chemotherapy	Toxicity
Recht/Bellon [1,2]	Rand 240 patients	RT-CT vs. CT-RT	CAMPF (4)	No difference
Serin [20]	Retro 154 patients	CT conc RT	Mitx + 5 FU + C (4–6)	G3 skin 4.5%
Bellon [21]	Retro 45 patients	CT conc RT	Taxanes	G3 skin 20% docetaxel
Burnstein [22]	Rand 40 patients	CT conc RT	Taxanes Weekly vs. 4 cycles	G3 pneumonitis 25% weekly
Sanguineti [23]	Prosp 47 patients	RT-CT vs. CT-RT	CEF 14 vs. CEF 21	No difference
Rouëssè [24]	Rand 638 patients	Conc RCT vs. seq CT-RT	C-Mitx-5FU vs. CEF	G3–4 leucopenia (A) Cardiac (B)
ARCOSEIN [25]	Rand 716 patients	CT-RT vs. Conc RCT	C-Mitx-5FU	No difference
Livi [26]	Prosp 60 patients	Conc RCT	AC/EPI + CMF	G3 skin 8.9%

Legend: Rand = randomized; Retro = retrospective; Prosp = prospective; seq CT-RT = Ct and sequential RT; RT-CT = Rt and sequential CT; Conc RCT = concurrent CT-RT.

**Table 2:** CMF or AC (concurrent vs. sequential RT).

Authors	Study	Sequencing	Chemotherapy	Toxicity
Faul [27]	Prosp 116 patients	CMF conc RT	st CMF vs. i.v. CMF	No difference
Fiets [28]	Prosp 112 patients	Conc CT	AC vs. CMF	High grade with AC
Arcangeli [29]	Rand 206 patients	Conc vs. Seq	i.v. CMF	No difference
Han [30]	Prosp 208 patients	Conc vs. Seq	i.v. CMF	No difference
Livi [31]	Retrosp 485 patients	RT seq CMF vs. CMF vs. RT	i.v. CMF	G2 skin in RCT
Ismaili [32]	Retro 244 patients	Conc CT	Ac-CT vs. CMF	G2-G3 skin/hematology in Ac-CT

Legend: st CMF = standard CMF; i.v. CMF = intravenous CMF; AC = adryamicin + cyclofosfamide; Ac = anthracycline-based.
